# Positive Effects of Probiotic Therapy in Patients with Post-Infectious Fatigue

**DOI:** 10.3390/metabo13050639

**Published:** 2023-05-08

**Authors:** Katharina Obermoser, Natascha Brigo, Andrea Schroll, Pablo Monfort-Lanzas, Johanna M. Gostner, Sabine Engl, Simon Geisler, Miriam Knoll, Harald Schennach, Günter Weiss, Dietmar Fuchs, Rosa Bellmann-Weiler, Katharina Kurz

**Affiliations:** 1 Department of Internal Medicine II, Medical University of Innsbruck, Anichstrasse 35, 6020 Innsbruck, Austria; katharina.obermoser@student.i-med.ac.at (K.O.); natascha.brigo@i-med.ac.at (N.B.);; 2 Institute of Medical Biochemistry, Biocenter, Medical University of Innsbruck, Innrain 80, 6020 Innsbruck, Austria; 3 Institute of Bioinformatics, Biocenter, Medical University of Innsbruck, Innrain 80, 6020 Innsbruck, Austria; 4 Institute of Hygiene and Medical Microbiology, Medical University of Innsbruck, Schoepfstrasse 41, 6020 Innsbruck, Austria; 5 Central Institute of Blood Transfusion and Immunology, University Hospital, Anichstr. 35, 6020 Innsbruck, Austria; 6 Institute of Biological Chemistry, Biocenter, Medical University of Innsbruck, Innrain 80, 6020 Innsbruck, Austria

**Keywords:** post-infectious fatigue, multi-strain probiotic, depression, quality of life, interferon-gamma mediated pathways

## Abstract

Post-infectious fatigue is a common complication that can lead to decreased physical efficiency, depression, and impaired quality of life. Dysbiosis of the gut microbiota has been proposed as a contributing factor, as the gut–brain axis plays an important role in regulating physical and mental health. This pilot study aimed to investigate the severity of fatigue and depression, as well as the quality of life of 70 patients with post-infectious fatigue who received a multi-strain probiotic preparation or placebo in a double-blind, placebo-controlled trial. Patients completed questionnaires to assess their fatigue (fatigue severity scale (FSS)), mood (Beck Depression Inventory II (BDI-II)), and quality of life (short form-36 (SF-36)) at baseline and after 3 and 6 months of treatment. Routine laboratory parameters were also assessed, including immune-mediated changes in tryptophan and phenylalanine metabolism. The intervention was effective in improving fatigue, mood, and quality of life in both the probiotic and placebo groups, with greater improvements seen in the probiotic group. FSS and BDI-II scores declined significantly under treatment with both probiotics and placebo, but patients who received probiotics had significantly lower FSS (*p* < 0.001) and BDI-II (*p* < 0.001) scores after 6 months. Quality of life scores improved significantly in patients who received probiotics (*p* < 0.001), while patients taking a placebo only saw improvements in the “Physical limitation” and “Energy/Fatigue” subcategories. After 6 months neopterin was higher in patients receiving placebo, while no longitudinal changes in interferon-gamma mediated biochemical pathways were observed. These findings suggest that probiotics may be a promising intervention for improving the health of patients with post-infectious fatigue, potentially through modulating the gut–brain axis.

## 1. Introduction

Following mainly viral infections, a substantial proportion of patients suffer from fatigue, which can be severe and last for weeks to months [1]. This is often associated with limitations in their physical ability, and some are even unable to work. This post-infectious fatigue is particularly well documented after infections with Epstein Barr virus (EBV), Dengue virus, and influenza [2,3,4,5]. Similar observations have been made after other viral diseases such as cytomegaly virus (CMV), parvovirus B19, human herpesvirus 6 (HHV-6), influenza, or adenovirus, and such infections have been proposed to trigger the development of myalgic encephalitis/chronic fatigue syndrome (ME/CFS) [6,7]. Similarly, following COVID-19 infection, many patients suffer from post-infectious fatigue, which is occasionally accompanied by dyspnoea, sleep disturbances, gastrointestinal symptoms, pain, cognitive impairment as well as depressed mood and symptoms of anxiety for several weeks to months [8,9,10,11,12].

Infection of gut epithelial cells with SARS-CoV2 has been shown to induce dysbiosis, intestinal inflammation, and gastrointestinal symptoms [13,14]. Furthermore, also an ongoing expression of SARS-CoV2 RNA and persistence of viral nucleocapsid protein in gut epithelia and CD8+ T cells has been demonstrated in many patients with inflammatory bowel disease (IBD) more than 7 months after mild acute COVID-19 infection [15]. Interestingly, post-acute sequelae of COVID-19 infection were described by most patients with viral antigen persistence in the gut, while patients without viral antigen persistence did not have such complaints [15]. Actually, dysbiosis of the gut microbiota has also been proposed earlier to contribute to the development of ME/CFS syndrome, however, data are not conclusive until now [16]. In fact, many patients with post-infectious fatigue also report gastrointestinal symptoms such as abdominal pain, constipation/diarrhea, or bloating, indicating irritable bowel syndrome (IBS) [17]. In a meta-analysis, significantly higher anxiety and depression levels have been described in IBS patients than in controls [18]. Our experience is that patients with post-infectious fatigue often suffer from depression, however, this has not been addressed in the literature thus far.

Dysbiosis of gut microbiota has been proposed to negatively affect physical and mental health via the gut–brain axis. Several studies have demonstrated the link between dysbiosis of gut microbiota, depression, and energy metabolism. For instance, Yu and co-workers 2017 found that depression is associated with significant changes in gut microbiota and fecal metabolic phenotype [19]. Other studies proposed that dysbiosis of gut microbiota leads to obesity and metabolic inflammation [20] and is associated with insulin resistance and increased food intake [21].

Moreover, there is reasonable evidence that the gut microbiota modulates behavior and brain processes, such as pain perception, stress response, and brain chemistry, despite the underlying molecular mechanisms remaining unclear [22,23,24]. Germ-free mice (without gut bacteria) exhibit increased stress response and anxiety-like behaviors, as well as deficits in social behavior and memory—all of which are common in depression [25]. Treatment with probiotics or restoration of commensal microbiota has reversed these depressive behaviors [25,26].

As probiotics have been shown to be useful in patients with depression (see also meta-analysis by [16]) and have been proposed as effective for the management of IBS and IBS-associated neurodegenerative and psychiatric comorbidities recently [27], we wanted to investigate, whether multi-strain probiotics are beneficial in patients with post-infectious fatigue. Furthermore, we wanted to determine the degree to which patients with post-infectious fatigue suffer from depression or impaired quality of life. Last, but not least, we aimed to explore whether alterations of immune activation and interferon-gamma (IFN-γ) mediated pathways- which have been shown earlier to be related to depression, impaired quality of life, and fatigue [28,29,30] might be involved. As immune-mediated alterations of tryptophan and phenylalanine metabolism can impair the formation of serotonin and dopamine [18,19,20], the relationship of amino acid metabolism disturbance with clinical symptoms of patients with post-infectious fatigue and effects of the intervention on these pathways were studied.

## 2. Materials and Methods

### 2.1. Study Population

For the present study, a total of 70 patients (46 female, 24 male) of the Department of Internal Medicine II, of the University Hospital Innsbruck, Austria were enrolled between January 2018 and March 2022. Patients who had been suffering from fatigue after an infection for longer than 4 weeks and who had no exclusion criteria (see below) were included. Of the 70 participants enrolled in the study, four participants dropped out before completing all three examinations, resulting in a completion rate of 94.3% (66 out of 70). The reasons for dropout of the study were: Noncompliance (n = 2), and the development of a new-onset depression or chronic fatigue syndrome (n = 2).

Patients with an acute infection, autoimmune disease, known immunodeficiency, chronic inflammatory bowel disease, liver disease, and renal insufficiency requiring dialysis were not included in the study. Moreover, patients with a prior diagnosis of depression or treatment with anti-depressants, chronic fatigue syndrome, and patients under immunosuppressive therapy or immunoglobulin substitution were excluded. In addition, patients taking probiotic dietary supplements during the last 2 months were not included in the study. Patients with more than 4 co-morbidities or 4 medications were excluded.

The participants were randomly assigned to receive either a multi-strain probiotic drug, “OMNi-BiOTiC^®^ STRESS Repair 9” (Institute AllergoSan, Graz, Austria), or placebo in form of corn starch for 6 months. The composition of “OMNi-BiOTiC^®^ STRESS Repair 9” is: Corn starch, maltodextrin, inulin, potassium chloride, vegetable protein (rice), 9 human bacterial strains, magnesium sulfate, fructooligosaccharides (FOS), enzymes (amylases), vitamin B2 (riboflavin 5′-sodium phosphate), vitamin B6 (pyridoxine hydrochloride), manganese sulfate, vitamin B12 (cyanocobalamin). For the bacterial strains, 7.5 × 10^9^ bacteria for 3 g were used. The 9 human bacterial strains were: *Lactobacillus casei* W56, *Lactobacillus acidophilus* W22, *Lactobacillus paracasei* W20, *Bifidobacterium lactis* W51, *Lactobacillus salivarius* W24, *Lactococcus lactis* W19, *Bifidobacterium lactis* W52, *Lactobacillus plantarum* W62 and *Bifidobacterium bifidum* W23. One sachet of OMNi-BiOTiC^®^ STRESS Repair 9 (=3 g) was mixed into approx. 1/8 L water 1–2 times daily on an empty stomach.

The study was designed as a prospective, double-blind placebo-controlled pilot study.

Moreover, the quality of life, immune status, a tendency to depression, and severity of fatigue were assessed by four questionnaires. The participants completed them independently. A total of three blood samples were taken at the time of study enrolment and subsequently after 3 and 6 months, between 8 and 12 am.

The study conformed to the ethical principles outlined in the Declaration of Helsinki and was approved by the ethical committee of Innsbruck Medical University (ID of the Ethical vote: EK Nr. 1158/2017). All patients signed a written informed consent after an in-depth explanatory interview before participating in the study.

### 2.2. Evaluation of Quality of Life, Depression, Fatigue, and Immune Status

#### 2.2.1. Quality of Life

For the objective assessment of the health-related quality of life, the subjects were given the “Questionnaire on the State of Health (SF-36)”. The SF-36 contains 36 questions that deliver information on the patient’s state of health. Statements are made regarding the general health perception, physical health, limited physical-related role function, physical pain, vitality, mental health, limited emotional, function, and social functioning.

For the evaluation of the SF-36 questionnaire, the average score of all questions was calculated. The possible score ranges from 0 to 100 points. A total of 0 points represent the greatest possible restriction of health, while 100 points represent no restriction at all. High values in the SF-36 describe a high quality of life and consequently the absence of health restrictions, low values indicate a high degree of health impairment.

#### 2.2.2. Depression

The BDI-II (Beck Depression Inventory, version II) was used to assess possible depression and, if available, the severity of the depression. Briefly, this involves 21 questions, each with four possible answers ranging from 0 (least severe) to 3 (strongest expression of the symptom). The questions or statements refer to how the patient felt during the last two weeks including the day on which the questionnaire is answered. By adding up the highest score in each category, a total score (0–63) is calculated. The five-level evaluation based on the total score obtained ranges from “No depression” (0–8 points) to the presence of a “Minimal depression” (9–13 points), a “Depression” (0–13 points), “Mild depression” (14–19 points), “Moderate depression” (20–28 points) and “Severe Depression” (29–63 points).

#### 2.2.3. Fatigue

To objectively assess the severity of fatigue in participants, the Fatigue Severity Scale (FSS) was used. Nine statements had to be rated on a scale from 1 to 7. 1 means “Do not agree with the statement at all” and 7 means “Agree completely”. The statements refer in each case to the feeling of fatigue during the previous week. The mean value was then calculated from the nine answers. A mean value of higher than 4 points was considered as greater fatigue. This value was set as a study by Hewlett et.al reported a mean ± SD score in healthy adults as 2.3 ± 0.7 points and means of 4.7 ± 1.5 points for patients with systemic lupus erythematosus and 4.2 ± 1.2 points for people with rheumatoid arthritis [31].

As some patients forgot or did not manage to bring their questionnaires to the appointments (2 at baseline, 7 at 3 months, 5 at 6 months; despite repetitive reminding of the study coordinator) only questionnaire data of patients who had filled at least 2 questionnaires over the course of the intervention were used for calculations to assess effects of the intervention on QoL, mood, and fatigue. Thus, data from 61 patients were analyzed (time point 6 months).

#### 2.2.4. Laboratory Measurements

Analyses of routine parameters and vitamin status in blood samples were performed at the Central Institute for Medical and Chemical Laboratory Diagnosis of the Innsbruck University Hospital. Neopterin and amino acid measurements were performed at the Institutes of Medical Biochemistry and Biological Chemistry, Biocenter, of the Medical University of Innsbruck. Neopterin (Neo) concentrations were measured by enzyme-linked immunosorbent assay (BRAHMS GmbH, Hennigsdorf, Germany) following the manufacturer’s protocol (sensitivity, 2 nmol/L). Concentrations of kynurenine (Kyn), tryptophan (Trp), phenylalanine (Phe), and tyrosine (Tyr) were analyzed in the patients’ sera via high-performance liquid chromatography, as described earlier [32,33]. After chromatographic separation on a LiChroCART RP-18 end-capped column (55-4, 3 µm, Merck, Darmstadt, Germany), the analytes Trp, Phe, and Tyr were measured via their native fluorescence (Trp: excitation wavelength of 286 nm, emission wavelength of 366 nm; Phe and Tyr: excitation wavelength of 210 nm, emission wavelength of 302 nm). Kyn and the internal standard, L-nitrotyrosine, were detected by UV-absorbance at a wavelength of 360 nm. The ratios of Kyn to Trp (Kyn/Trp) and Phe to Tyr (Phe/Tyr) were calculated as surrogates of indoleamine 2,3-dioxygenase and phenylalanine hydroxylase activity, respectively [33,34]. Results of amino acid measurements were compared to values of age-matched healthy blood donors (42 healthy controls; 29 females with a median age of 44; 13 males with a median age of 39) [35].

### 2.3. Statistical Analysis

Statistical analysis of the data was performed using the programs IBM SPSS Statistics Version 28 (IBM Corporation, Armonk, NY, USA). *p*-values lower than 0.05 were considered statistically significant. The Kolmogorov-Smirnov test showed that most of the data were not normally distributed. Therefore, non-parametric tests such as the Mann–Whitney U test were applied where necessary. For a longitudinal analysis over the course of the treatment, a two-way ANOVA with Tukey post hoc tests was used. For the analyses conducted at specific time points, missing data from subjects were treated as missing values, without any attempt to fill in or estimate the absent information.

## 3. Results

### 3.1. Study Population

The study consisted of 46 (65.7%) women and 24 (34.3%) men, with a median age of 40 (19–81 years). Examination of the general characteristics of the study population revealed a significant age difference between the median age of women and men (43 years vs. 34 years; *p* = 0.026). A total of 20 patients reported prior infection with Epstein Barr virus (EBV)- virus (12 male and 8 female), and 14 (9 female and 5 male) suffered from fatigue after COVID-19 infection. Four women and seven men stated that they had a history of borreliosis, while most patients had experienced previous respiratory tract infections before the development of fatigue.

Thyroid disease was found in 28.3% of the women and 12.5% of the men (n.s.). Among them, 17.4% of women and 1 man (4.2%) had Mb. Hashimoto (n.s.). Muscle pain was reported by 30.4% of women and 37.5% of men (n.s.). Before the start of the study, 17.4% of women (n = 8) and 20.8% of men (n = 24) experienced insomnia (n.s.). A total of 51.5% (n = 35) of all study participants had allergies, including 23 women and 12 men. Pollinosis was the most common allergy, affecting 35.9% (n = 23) of participants.

### 3.2. Fatigue

**Figure 1:**
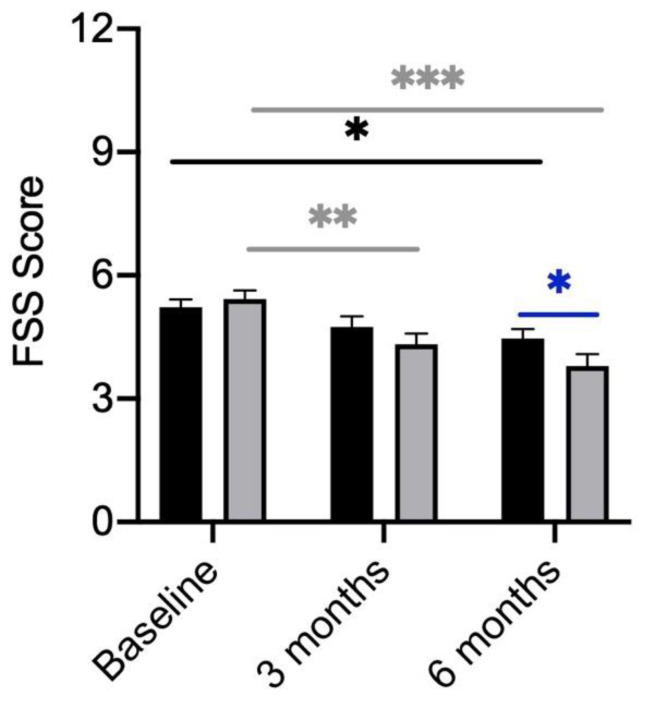
FSS Scores of participants divided by treatment (verum; grey column) with placebo (black column). FSS scores compared between the verum and placebo arms over the course of treatment. Significant differences were determined by Mann–Whitney U test. Grey * shows significant differences between patients of the verum group at different time points. Black * shows significant differences between patients of the placebo group at different time points. Blue * shows significant differences between placebo and verum. * *p*-value < 0.05; ** *p*-value < 0.01; *** *p*-value < 0.001.

Fatigue was defined as a Fatigue Severity Scale (FSS) score of higher than 4 points. Among the 70 study participants, 55 patients (78.6%) fulfilled this criterion. The mean score of all subjects was 5.4 (±1.2) points. Men and women both presented with a mean score of 5.4 (±1.2). No significant gender differences could be seen at baseline. In addition, FSS scores did not differ between participants receiving the placebo and participants receiving the probiotic. (Figure 1). Over the course of treatment, a significant reduction in the FSS score in the verum and placebo group became evident (Figure 1). However, the participants receiving the probiotic showed a significantly higher reduction in their FSS score after 6 months (Figure 1).

Interestingly, after 6 months of probiotic or placebo treatment, a total of 21 participants improved (34.4%), of which 15 were in the verum arm (71.4%) and 6 were in the placebo arm (28%). A total of 39 of all subjects achieved the same result (63.9%) and only one person worsened, who was in the placebo arm. Treatment with the probiotic thus led to a significant improvement in FSS-scores over the course of 6 months compared to patients receiving the placebo (*p* = 0.024).

### 3.3. BDI-II

**Figure 2:**
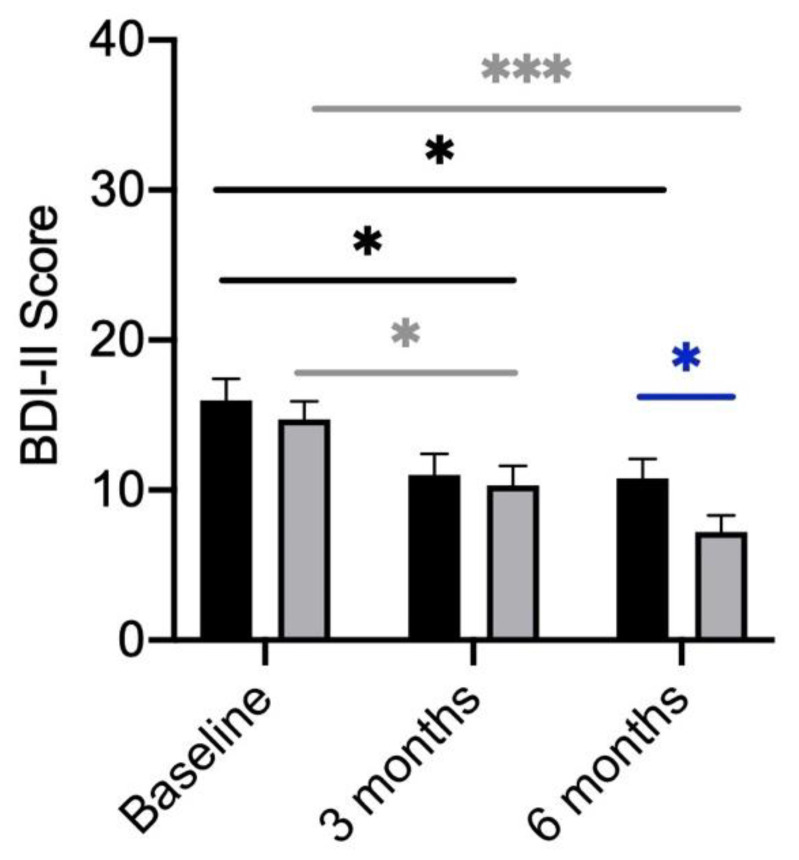
BDI-II Scores of participants divided by treatment (verum; grey column) with placebo (black column). BDI-II scores compared between the verum and placebo arms over the course of treatment. Significant differences determined by Mann–Whitney U test. Grey * shows significant differences between patients of the verum group at different time points. Black * shows significant differences between patients of the placebo group at different time points. Blue * shows significant differences between placebo and verum.* *p*-value < 0.05; *** *p*-value < 0.001.

The BDI-II questionnaire was used to objectively assess the subjects’ tendency towards depression. Scores ranging from 2 to 33 points (maximum achievable score: 63) were obtained with a mean of 15.73 (±7.52). Women scored a mean of 15.36 (±7.97) and men 16.43 (±6.68) at baseline. In addition, no significant differences in BDI-II scores were detected between the placebo and verum groups (Figure 2).

A considerable number of patients experienced depression at baseline: minimal (n = 15, female: 7; male: 8), mild (n = 19, female: 11; male: 8), moderate (n = 18, female: 14; male: 4), severe (3 women).

In the longitudinal comparison over the three time points, both in the verum group (*p* ≤ 0.001) as well as in the placebo group (*p* = 0.05) significantly lower BDI-II scores were reached (Figure 2). Even though both groups showed an improvement in their BDI-II scores, the participants receiving the probiotic showed a significantly higher reduction after 6 months (Figure 2).

Overall, after 6 months, 27 (44.3%) participants showed an improvement in the degree of depression, 16 participants receiving the probiotic and 11 receiving the placebo. The BDI-II score remained the same for 30 (49.1%) of the participants, 11 in the verum group and 19 in the placebo group. A worsening was observed in four participants, one in the group receiving the probiotic and three receiving the placebo. Between the two study arms “verum” and “placebo” significant differences were found for the change in the depression severity (Figure 2). Treatment with probiotics led to a higher reduction of BDI-II scores compared to placebo-treated participants.

### 3.4. Quality of Life

**Figure 3:**
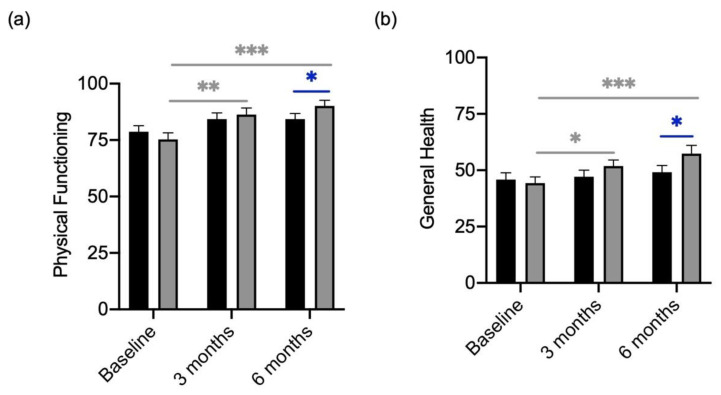
Quality of Life Scores in “Physical functioning” and “General Health” of participants divided by treatment (verum; grey column) with placebo (black column). (**a**) Changes in “Physical functioning” scores over the course of treatment with placebo and verum. (**b**) Changes in “General Health” scores over the course of treatment with placebo and verum. Grey * shows significant differences between patients of the verum group at different time points. Blue * shows significant differences between placebo and verum. * *p*-value < 0.05; ** *p*-value < 0.01; *** *p*-value < 0.001.

The results of the QoL questionnaire showed that the participants felt restricted in the dimension of “Physical Functioning” with an average of 76.07 (±18.12; Figure 3a). Over the course of the treatment with the probiotic a significant improvement in “Physical Functioning” was determined, in the placebo group only little increases were seen (Figure 3a). Similar results were observed in the subcategory of “General Health”. At baseline, patients of the verum arm and placebo arm showed rather low levels (Figure 3b). Supplementation with the probiotic showed a significant increase in the “General health” score, which was not seen in the group receiving the placebo (Figure 3a,b).

**Figure 4:**
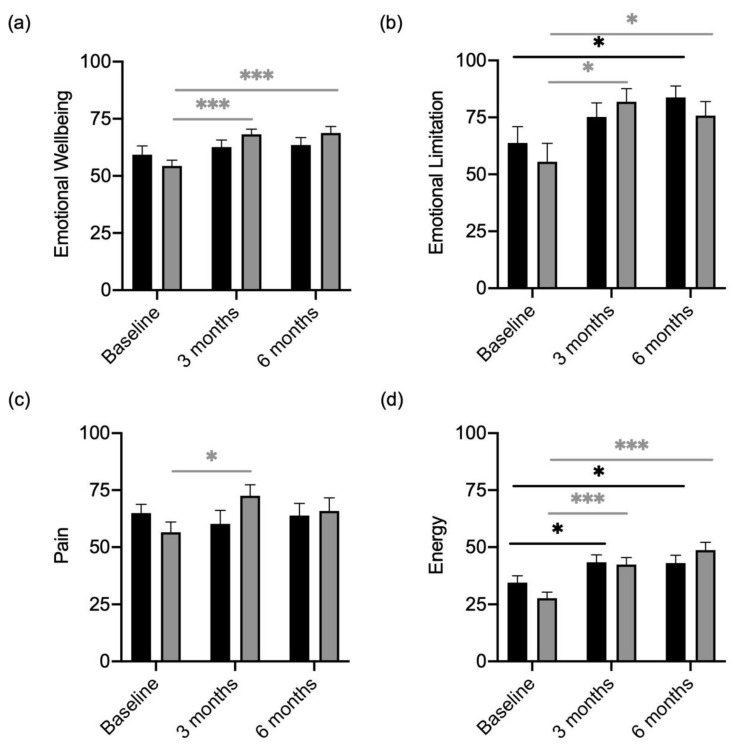
Quality of Life Scores in “Emotional Limitation”, “Emotional Wellbeing”, “Pain” and “Energy” of participants divided by treatment (verum; grey column) with placebo (black column). (**a**) “Emotional Wellbeing” scores compared between verum and placebo arms over the course of treatment. (**b**) “Emotional Limitation” scores compared between verum and placebo arms over the course of treatment. (**c**) “Pain” scores compared between verum and placebo arms over the course of treatment. (**d**) “Energy” scores compared between verum and placebo arms over the course of treatment. Grey * shows significant differences between patients of the verum group at different time points. Black * shows significant differences between patients of the placebo group at different time points. * *p*-value < 0.05; *** *p*-value < 0.001.

Furthermore, in the dimensions of “Emotional Limitation”, “Emotional Wellbeing” “Pain” and “Energy”, baseline scores were similar in the placebo and probiotic participants (Figure 4a–d). Participants receiving the probiotic presented with higher scores for “Emotional Limitation” (Figure 4b) and “Emotional Wellbeing” (Figure 4a) over the course of 6 months. “Pain” also improved in the verum group from baseline to 3 months (Figure 4c). Both, participants receiving the placebo and the verum reported a decrease in health restrictions in the category “Emotional Limitation” (Figure 4b) and “Energy” (Figure 4c).

**Figure 5:**
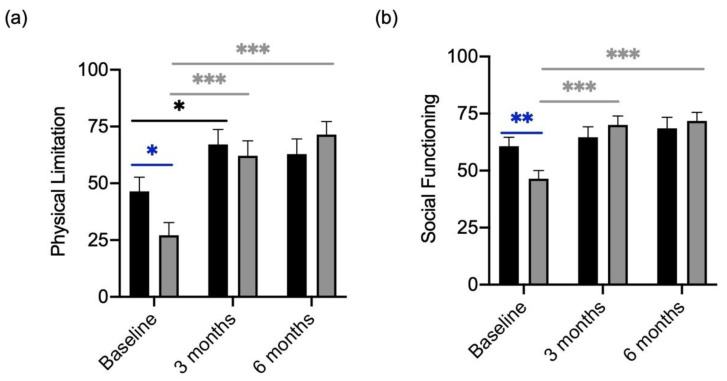
Quality of Life Scores in “Physical Limitation” and “Social Functioning” of participants divided by treatment (verum; grey column) with placebo (black column). (**a**) “Physical Limitation” and scores compared between the verum and placebo arm over the course of treatment. (**b**) “Social Functioning” scores compared between the verum and placebo arm over the course of treatment.Significant differences were determined by Mann–Whitney U test. Grey * shows significant differences between patients of the verum group at different time points. Black * shows significant differences between patients of the placebo group at different time points. Blue * shows significant differences between placebo and verum. * *p*-value < 0.05; ** *p*-value < 0.01; *** *p*-value < 0.001.

At baseline participants who received the probiotic, had worse scores compared to patients consecutively receiving the placebo in the categories “Physical Limitation” and “Social Functioning” (Figure 5a). The scores for “Physical Limitation” and “Social Functioning” increased significantly in the group receiving the probiotic (Figure 5b) and slightly in the placebo group. After 6 months of treatment, both scores were similar in participants receiving the placebo and participants receiving the probiotic (Figure 5).

Results of the SF-36 subcategories showed that treatment with a probiotic over the course of 6 months significantly improved the quality of life compared to participants receiving the placebo.

### 3.5. Subjective Improvement of Digestion over the Course of Treatment

In the case histories of the respective follow-up after 3 and 6 months, all study participants were asked for the subjective condition of their digestive function. At baseline, 25 participants stated that they had no problems with their digestive system. Otherwise, diarrhea (eight participants), constipation (eleven participants), flatulence (five participants), and abdominal pain (three participants) were the most common digestive problems. Seventeen participants complained about several digestive problems.

According to the participants, digestion improved after 3 months in 21 participants in the verum group and in 19 participants in the placebo group (n.s.). Thirty female participants and twelve male participants felt improvement after 3 months (n.s.). Between the groups “verum” and “placebo”, no significant difference was observed. Thus, digestion improved in 22 of the participants taking a placebo preparation. In comparison, digestion was improved in 21 in the verum group (n.s.).

### 3.6. Evaluation of the Immune Activation Parameters and Immune-Mediated Changes in Tryptophan and Phenylalanine Metabolism

Serum markers of inflammation were within normal ranges in most patients. The acute phase protein CRP was elevated in only two of the 70 study participants. In the group with diagnosed fatigue (i.e., FSS-score > 4, mean CRP 0.10 mg/dL) as well as in the group with lower FSS-scores (0.45 mg/dL) the values did not differ. The blood sedimentation rate was in the normal range in all the study participants. Female participants showed lower hemoglobin and decreased hematocrit levels and erythrocytes compared to male study participants. Initially, three participants had iron deficiency, which included two females (2.86%) and one male (1.43%). Moreover, at the beginning of the study, four participants (5.71%) had a folic acid deficiency, of which three were women and one was a man. Vitamin D deficiency was encountered frequently (47.14%). Interestingly, 54 participants (77.14%) had elevated thiamine levels, and also vitamin B6 was elevated in 13 cases (18.57%). B vitamins were supplemented by 28.57% of the participants (n = 20).

The immune activation parameters neopterin and kynurenine, as well as amino acid concentrations tryptophan, tyrosine, and phenylalanine, were compared with values of healthy age-matched blood donors as a reference. When analyzing the IFN-γ-induced metabolic pathways, all parameters except the ratio of Kyn to Trp differed compared to the age-matched healthy control group (42 healthy controls; 29 females with a median age of 44; 13 males with a median age of 39) at baseline (Table 1). A significant difference was found between men and women for tryptophan (*p* = 0.013), kynurenine (*p* = 0.04), tyrosine (*p* = 0.03), and the Phe/Tyr ratio (*p* = 0.035). All of the above values, except for the Phe/Tyr ratio, were higher on average in males compared to females.

The concentrations of all investigated laboratory parameters remained relatively stable during the course of treatment. Women and men did not differ regarding IFN-γ-induced metabolic pathways metabolites after 6 months of therapy, except for tryptophan (see Table 2).

The investigated parameters did not differ between verum and placebo-treated groups after 3 months of treatment. Additionally, in the longitudinal analyses, the parameters did not change significantly after 6 months of treatment, neither with verum nor placebo. However, after 6 months, the inflammatory parameter neopterin was significantly higher in the placebo arm (*p* = 0.026), and kynurenine levels also tended to be higher (*p* = 0.077), when parameters were compared by the Mann–Whitney U test.

## 4. Discussion

This study shows that patients with post-infectious fatigue are very often affected by depression and at the same time experience restrictions in their quality of life and subjective performance. This finding is important, because these restrictions often limit the ability of young patients to work, especially if they develop myalgic encephalitis/chronic fatigue syndrome (ME/CFS).

Earlier studies suggested that increased immune activation, oxidative stress, and impaired neurotransmitter synthesis play a role in patients with ME/CFS [36]. Additionally, immune-mediated changes in tryptophan and phenylalanine metabolism were associated with impaired quality of life, fatigue, and depression in patients with cancer and infections [28,29,30,37,38]. We, therefore, wanted to investigate whether biochemical alterations might also be involved in patients with post-infectious fatigue and whether intervention with a multi-strain probiotic is effective to improve patients’ symptoms.

Compared to age-matched healthy blood donors we found slightly higher neopterin and phenylalanine concentrations, while tryptophan, kynurenine, and tyrosine levels were lower. Concentrations of amino acids tryptophan and tyrosine as well as kynurenine concentrations differed between women and men with post-infectious fatigue at baseline, while after 6 months of intervention, only tryptophan concentrations still differed. Rather unexpectedly, treatment with probiotics (and also placebo) did not change the concentrations of the investigated parameters significantly, and with longitudinal tests no change over time was seen. Still, the finding, that neopterin levels were higher (and kynurenine levels tended to be higher) in patients with placebo compared to verum after 6 months suggests that long-term intake of probiotics might also influence immune cascades- but only slightly and dependent on individual circumstances.

Overall, the intervention was very effective to improve patients’ fatigue, mood, and QoL: In both the verum and placebo arms, the BDI-II scores and FSS scores were significantly lower after six months. Effects of probiotics were superior to placebo: After 6 months 15 patients in the verum group had significantly less fatigue, while only 6 patients improved in the placebo arm. Additionally, BDI II scores were significantly lower in patients under treatment with probiotics compared to placebo after 6 months, fewer patients showed scores indicating depression than at baseline. Patients receiving the verum also reported a significantly improved quality of life in all categories after 6 months, except for the category pain. Patients treated with placebo only improved regarding energy/fatigue, additionally, they also had higher scores for emotional limitations after 6 months, and for physical limitations after 3 months; however, this effect was not seen anymore after 6 months. Patients taking probiotics on the other hand reported improved general health, physical functioning, and emotional well-being, as well as less fatigue, and social and physical limitation.

The improvements in the FSS and BDI II scores in the placebo arm cannot be clearly attributed: Our impression was that the intervention was helpful for the patients because their problems (fatigue/impaired physical, emotional, and social functioning) were taken seriously (despite “normal laboratory parameters” in a standard lab). Moreover, patients appreciated the opportunity to actively contribute to their own “recovery” by participating in the study. Most patients, who had been highly efficient prior to the infection, expressed feelings of helplessness and subsequent depression, as they were repeatedly told to be “physically healthy” (despite their differing perceptions). The majority of patients were more inclined to believe that metabolic changes caused by the infection, rather than “hidden psychological stress”, were responsible for their fatigue. Many also suspected that repeated antibiotic treatments had negatively affected their digestive function.

In fact, over half of the patients experienced symptoms of irritable bowel syndrome (bloating, abdominal pain, nausea, constipation, or diarrhea), and numerous patients noticed an improvement in their digestion during the intervention. Patients were also encouraged to follow a healthy Mediterranean diet, and specific vitamin deficiencies (particularly vitamin D deficiency) were addressed. This approach motivated patients to consistently take the study medication under the close guidance of an experienced therapist. As a result, compliance was exceptionally high, with only a few patients missing the 6-month check-up. The Mediterranean diet has previously been demonstrated to help alleviate symptoms of depression [39]. Additionally, also other factors might have been involved: Moss-Morris et al. had already investigated earlier that cognitive coping with illness can affect symptoms of exhaustion. Thus, patients with a strong focus of attention on physical sensations complain on average about significantly stronger fatigue symptoms and feel subjectively more affected by it [40]. In particular, catastrophizing cognitive processing of fatigue is associated with greater symptom expression and impairment [41]. In this respect, it could be assumed that cognitive behavioral therapy would be helpful for the patient. However, a study from the United Kingdom showed that 73% of the participants had no improvement in fatigue under cognitive behavioral therapy, 8% had an improvement and 14% had a worsening of fatigue [42]. Our intervention thus was more effective compared to cognitive behavioral treatment: 21 patients improved significantly regarding their fatigue, and most of them (15 patients) had been treated with the verum. 51.2% response in the verum group compared to 18.8% in the placebo arm indicates that the multi-strain probiotics were effective to alter metabolic alterations underlying the fatigue of patients-maybe by improving the diversity of gut microbiota, enhancing nutrient uptake and/or reducing the production of toxic metabolites produced by gut pathogens.

The finding that the mood improved in many patients during the intervention fits well with data from a meta-analysis, which demonstrated that probiotics are effective for the treatment of depression [16]. As in our study, the effect of a multi-strain probiotic preparation containing 9 bacterial strains on fatigue, mood, and quality of life in patients with post-infectious fatigue was investigated, we cannot make conclusions, about which single bacterial strains were most effective in improving the mood, QoL and fatigue of patients. Previous research has suggested that different probiotic strains may have distinct effects on gut microbiota and health outcomes. For example, *Lactobacillus plantarum* has been shown to improve symptoms of abdominal pain and bloating in IBS patients [43], while *Bifidobacterium lactis* was associated with improved gut barrier function [44,45,46]. An oral probiotic strain of *Lactobacillus rhamnosus* has been found to alter the level of gamma amino butyric acid (GABA) in brain regions related to emotional processing, resulting in reduced anxiety and depression-like behavior in BALB/c mice [22]. Additionally, depressive-like behavior in rats was markedly reduced by multi-strain probiotic treatment independent of diet [47]. Furthermore, in a randomized, double-blind, placebo-controlled clinical trial, patients receiving a probiotic capsule of *Lactobacillus acidophilus*, *Lactobacillus casei*, and *Bifidobacterium bifidum* reported a decrease in Beck Depression Inventory scores (BDI) compared to the placebo group [48].

However, not all studies indicate that probiotics are effective in treating depression. Romijn. et al. reported that treatment with *Lactobacillus helveticus* and *Bifidobacterium longum* did not improve patients’ depressive status. However, they point out that recruiting a patient cohort with mild, acute symptoms of low mood, compared to a patient group with a history of treatment resistance, may well result in a very different outcome [49]. It is important to note that studies with differing methodologies (e.g., probiotic dose and strains) and those conducted in populations without clinically diagnosed depression may account for the foregoing discrepancy in the literature [50,51]. Additionally, in previous clinical trials, improper dosing may have contributed to negative results, so optimal therapeutic dose ranges and durations should also be defined [52]. Earlier, a probiotic supplement was even proposed to be a more effective treatment for depression than conventional treatments (e.g., pharmacological therapies, brain stimulation therapies) without causing any notable adverse effects in humans or animals [53]. Unfortunately, there are no trials where probiotics and antidepressants were compared versus each other (at least to our knowledge)-which might, in fact, be interesting.

In patients with a placebo, the change in diet and the intake of vitamin D may have played a role, as 11 patients in this arm stated that they ate more vegetables, and reduced the intake of white flour products, sweets, and red meat. Already earlier it has been shown that fatigue and depression can both be influenced positively by changes in diet [54]. Thus, this strategy could be suggested to motivate patients before prescribing antidepressants. As the Mediterranean diet is also known to have anti-inflammatory effects [54], inflammatory processes might be modulated by dietary changes- however, at least in our population, we did not see strong effects on T helper cell type 1 immune activation (as reflected by neopterin concentrations) or immune-mediated tryptophan catabolism.

Further studies investigating changes in the gut microbiota or metabolites such as short-chain fatty acids, serotonin, or gamma amino butyric acid (GABA) during a probiotic intervention might be useful to investigate, which alterations are responsible for the positive effects on fatigue, mood, and quality of life in our pilot study. In fact, probiotic treatment might influence one or all of these suggested pathways-depending on the individual microbiota composition differently. Individualized probiotic therapy might therefore be an interesting option in patients with IBD and post-infectious fatigue.

Our study presents a few limitations. Firstly, it was executed as a single-center study, resulting in a limited sample size for our cohort. Secondly, there were notable differences between the verum and placebo-treated groups at baseline in two categories of the SF-36 questionnaire. Another limitation is that not all patients managed to bring their questionnaires at all three time points, which could have biased our data.

To summarize, we performed a placebo-controlled double-blind randomized trial, which investigated a very common, but so far “untreatable” disease. The results are promising and should prompt further longitudinal studies with more patients to investigate underlying pathomechanisms and changes in the microbiome of post-infectious fatigue patients. Especially in patients with Post-COVID19 conditions such studies will be helpful, as results of a recent study showed that the intervention with probiotics in combination with enzymes [55] or phyto preparations [56] was effective to reduce persistent symptoms and probiotic therapy during acute infection led to fewer symptoms compared to patients without such a treatment [57].

## Figures and Tables

**Table 1:** Mean ± SD of IFN-γ-induced metabolic pathways metabolites at baseline.

	Mean ± SD Healthy Age-Matched Control Group	Mean ± SD Study Population	*p*-Value (1)	Mean ± SD Female	Mean ± SD Male	*p*-Value (2)
Neopterin (nmol/L)	6.0 ± 1.6	7.3 ± 2.8	0.04	6.9 ± 2.7	7.0 ± 3.0	n.s.
Tryptophan (μmol/L)	68.4 ± 11.8	56.6 ± 9.7	0.005	55.6 ± 8.9	61.4 ± 9.5	0.01
Kynurenine (μmol/L)	1.8 ± 0.4	1.6 ± 0.4	0.03	1.5± 0.3	1.8 ± 0.3	0.04
Kyn/Trp (μmol/mmol)	27.2 ± 6.8	29.0 ± 6.4	n.s.	28.8 ± 6.9	29.8 ± 7.6	n.s.
Tyrosine (μmol/L)	87.8 ± 24.7	64.8 ± 16.4	0.004	62.0 ± 15.2	67.5 ± 13.1	0.03
Phenylalanine (μmol/L)	64.2 ± 10.6	81.0 ± 12.0	<0.001	78.4 ± 12.3	79.2 ± 12.4	n.s.
Phe/Tyr (μmol/μmol)	0.8 ± 0.1	1.3 ± 0.2	<0.001	1.3 ± 0.2	1.2 ± 0.2	0.03

(1) Significant differences between the healthy age-matched control group and study population. (2) Significant differences between females and males

**Table 2:** Mean levels ± SD of IFN-γ-induced metabolic pathways metabolites after 6 months of treatment.

	Mean ± SD Female 3 Months	Mean ± SD Male 3 Months	*p*-Value (1)	Mean ± SD Female 6 Months	Mean ± SD Male 6 Months	*p*-Value (2)
Neopterin (nmol/L)	7.3 ± 2.8	7.6 ± 4.4	n.s.	9.7 ± 13.3	7.7 ± 3.6	n.s.
Tryptophan (μmol/L)	55.5 ± 8.0	61.7 ± 13.1	0.007	46.9 ± 36.3	52.5 ± 40.2	0.04
Kynurenine (μmol/L)	1.6 ± 0.4	1.8 ± 0.4	0.03	1.7 ± 0.5	1.9 ± 0.4	n.s.
Kyn/Trp (μmol/mmol)	28.6 ± 6.4	29.6 ± 7.6	n.s.	30.3 ± 8.5	30.2 ± 6.6	n.s.
Tyrosine (μmol/L)	62.8 ± 17.7	68.3 ± 17.3	n.s.	60.0 ± 12.6	61.8 ± 14.0	n.s.
Phenylalanine (μmol/L)	77.8 ± 11.8	77.6 ± 13.3	n.s.	76.7 ± 13.9	77.9 ± 10.8	n.s.
Phe/Tyr (μmol/μmol)	1.3 ± 0.2	1.2 ± 0.2	0.04	1.3 ± 0.2	1.2 ± 0.2	n.s.

(1) Significant differences between females and males after 3 months of treatment. (2) Significant differences between females and males after 6 months of treatment.

## Data Availability

Results presented within this study are available within the manuscript. Blinded raw data are available upon request.
